# Synchronously diagnosed pre-sacral neurofibroma and cutaneous spitzoid melanoma: a fortuitous association?

**DOI:** 10.1186/1477-7819-2-31

**Published:** 2004-09-13

**Authors:** Oluwole Fadare, Denise Hileeto

**Affiliations:** 1 Department of Pathology, EP 2-631, Yale-New Haven Hospital, 20 York Street, New Haven, CT 06504, USA

## Abstract

**Background:**

At a U.S prevalence of 1 in 3000, Neurofibromatosis type-1 (NF-1) is a relatively common disorder. Amongst a variety of others, occurrence of 2 or more neurofibromas in the same patient represents one of the major diagnostic criteria for this disorder. Rarely, ocular, cutaneous or anorectal malignant melanomas may be identified in patients with NF-1, This rare association has caused controversy as to whether patients with NF-1 have an inherently higher risk for melanomas or whether the associations can be explained by chance alone.

**Case presentation:**

The purpose of this report is to highlight the unusual confluence of rare clinicopathologic features in a patient without NF-1. The patient was diagnosed with an 8.5 cm pre-sacral neurofibroma and was shortly thereafter diagnosed with a cutaneous malignant melanoma showing spitzoid features. Pre-sacral neurofibromas are rare in patients without NF-1; likewise, malignant spitzoid melanoma, a controversial histopathological entity, is distinctly uncommon.

**Conclusions:**

The synchronous diagnoses of these neural crest derived tumor entities in a patient without neurofibromatosis lends credence to the view that when these two lesions occur in patients with NF-1, the association is  coincidental.

## Background

Neurofibromatosis type-1 (NF-1) is a common autosomal dominant disorder characterized by multiple neurofibromas, *café-au-lait *spots, freckling of the inguinal or axillary regions, gliomas, iris hamartomas, and malignant peripheral nerve sheath tumors [1,2]. Neurofibromas are typically well-delineated and are composed of an admixture of various cell types, such as Schwann cells, fibroblasts and perineural-like cells and cells showing intermediate features [1,2]. Although as outlined above, multiple neurofibromas are characteristic of patients with NF-1, however, most cases of neurofibroma which are diagnosed in general are sporadic in nature. The vast majority of neurofibromas are cutaneous and less commonly are intraneural, within the soft tissues or viscera. Presacral neurofibromas or neurofibromas with presacral involvement are uncommon in patients without NF-1, and have been the subject of sporadic case reports over the past half-century [3-16]. Likewise, spitzoid melanoma or melanomas showing spitzoid-like features form only a small percentage of all malignant melanomas. This diagnosis is based on the rare finding that some melanomas displays cytologic features that are similar to those identified in the benign Spitz nevus [17]. The controversy associated with this lesion stems from the fact that some dermatopathologists do not believe in its existence and prefer to designate melanocytic proliferations meeting traditional criteria for malignancy as malignant melanomas, irrespective of the Spitzoid features [18]. To our knowledge, a synchronous presacral neurofibroma and cutaneous spitzoid melanoma have never been reported in a patient without neurofibromatosis. More importantly, the absence of NF-1 in our patient may have implications for the potential association between malignant melanoma and NF-1.

## Case presentation

**Figure 1:**
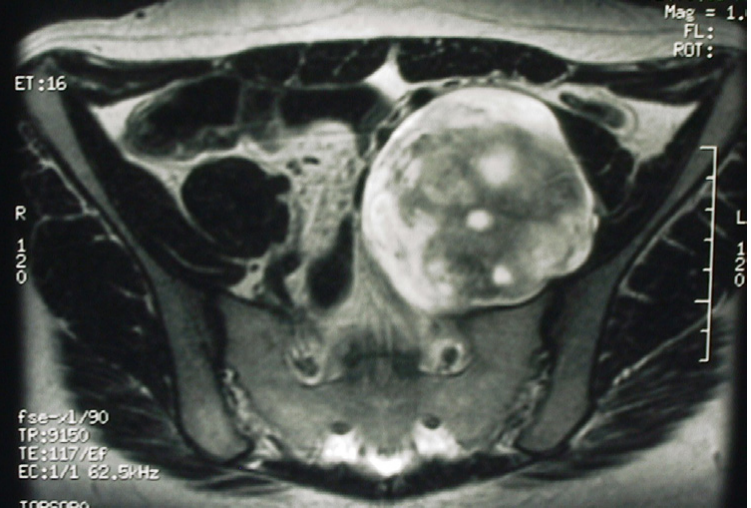
Computed tomographic scan of the pelvis showing a large, well-circumscribed presacral mass

In September 2000, a 35-year-old female without any history or clinical stigmata of NF-1 presented to her primary physician with complaints of a dull, localized left upper leg pain of several months' duration. An abdominal mass was palpated during a physical examination. Magnetic resonance imaging (MRI) as well as a computed tomographic (CT) scan of the abdomen and pelvis showed a large well-defined, near spherical mass in the left false pelvis which enhanced heterogeneously at a mean Hounsfield value of 44 units (Figure 1). The mass displaced the external iliac vein medially and psoas muscle laterally. It also abutted the upper surface of the left ovary without truly invading any of these or other surrounding structures. However, the mass was believed to be in the course of the left genitofemoral nerve and lumbar plexus. The decision was made to resect the mass. Intraoperatively, the tumor's capsule was found to be densely adhered medially to the external iliac vessels, with at least 10 external venous branches directly supplying the tumor. The tumor was carefully marsupialized out of the retroperitoneal area and the decision was made to leave the residual capsule, since an attempt at its removal would have entailed a highly morbid procedure that was not felt to be justified based on the histopathologic appearance of the tumor on frozen sections. Intraoperatively, a pigmented macular lesion with faintly irregular edges was noted in the left upper thigh, which was biopsied. Pathologic examination showed a malignant melanoma with spitzoid features. The precise circumstances regarding the duration of the lesion and whether there had been any increase in its size was unclear. She subsequently underwent a wide local excision (4 × 12 cm skin ellipse was removed) and sentinel lymph node biopsy, both of which showed no residual melanoma. The patient's postoperative course over the subsequent 2 years was remarkable for a relatively slow but progressive improvements in the neurologic symptoms related to her surgery. However, she showed no evidence of either tumor recurrence at last follow-up, 26 months postoperatively.

## Pathologic findings

**Figure 2:**
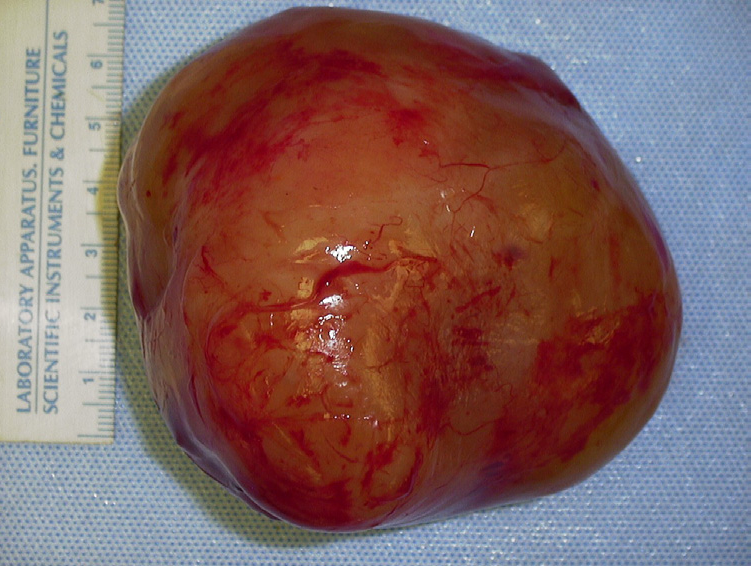
Macroscopic appearance of the external surface of the presacral mass

**Figure 3:**
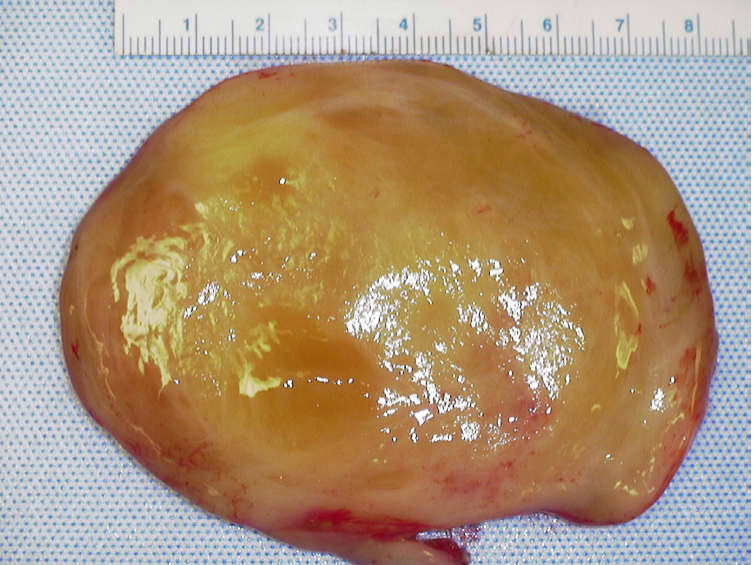
The cut surface of the presacral mass showing glistening myxoid, tan-yellow appearance

**Figure 4:**
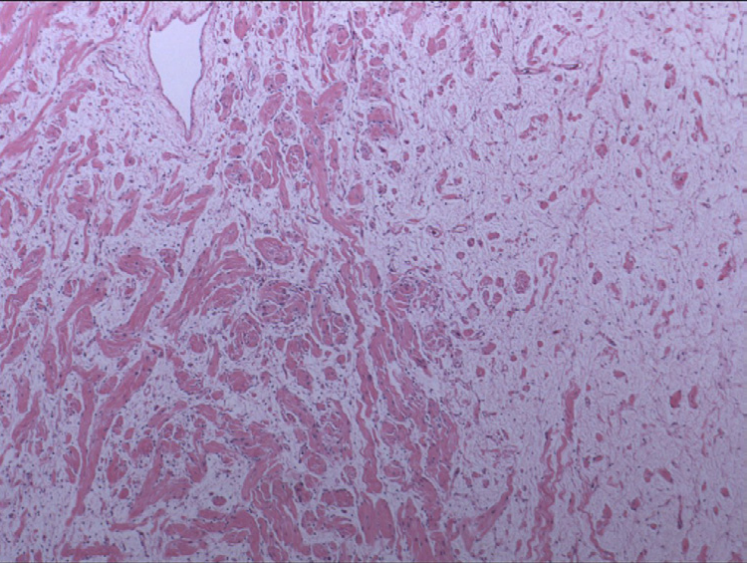
Microscopic appearance of the presacral mass showing a haphazard admixture of wavy Schwann cells and collagen fibers dispersed in a mucopolysaccharide matrix (Hematoxylin and Eosin, 20×)

**Figure 5:**
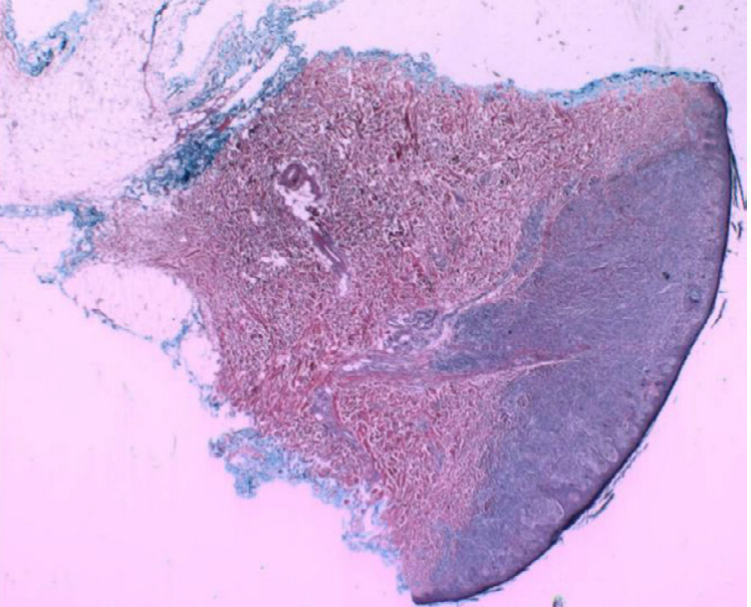
Photomicrographic panoramic view of the patient's cutaneous biopsy showing an asymmetric lesion with a basal confluent growth (Hematoxylin and Eosin 2×)

**Figure 6:**
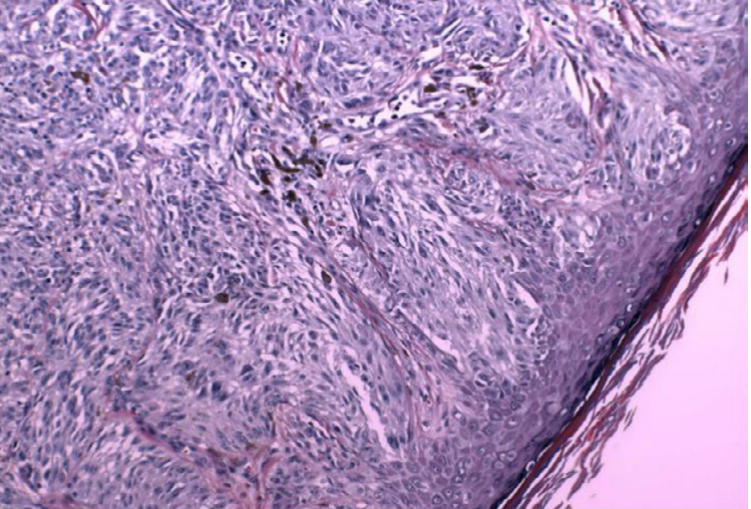
Photomicrograph of the junctional component of the tumor showing Spitzoid features of the lesional cells. Note that the junctional nests do not display a uniform vertical orientation towards the epidermis, as is expected in most Spitz nevi. (Hematoxylin and Eosin 40×)

**Figure 7:**
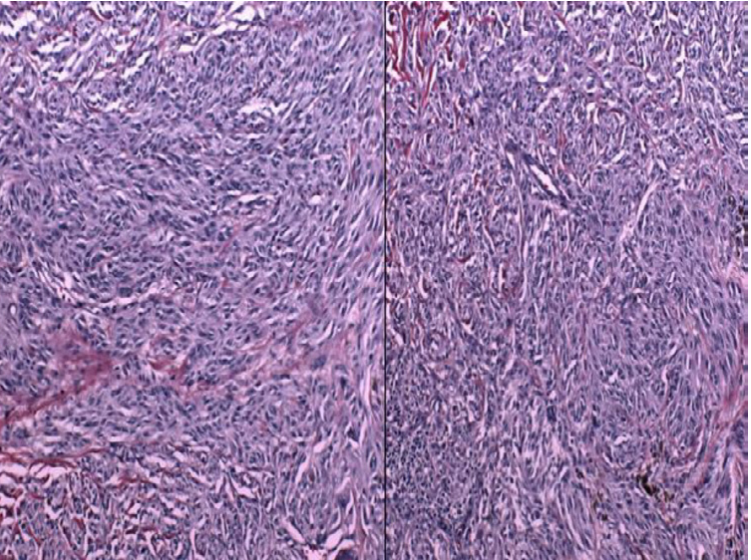
Interemediate-power view of the cutaneous lesional cells, showing the admixture of spindle and epithelioid cells (Hematoxylin and Eosin 20×)

**Figure 8:**
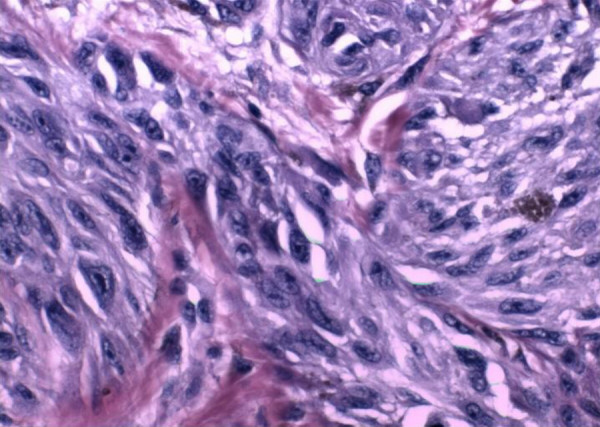
Photomicrograph showing the cytologic features of the lesion. Note the nuclear pleomorphism and prominent nucleoli. This focus was near the deep edge of the lesion, reflecting a lack of histological maturation (Hematoxylin and Eosin 20×)

The resected mass was spherical, weighed 270 grams and measured 8.5 × 7 cm × 7 cm (figure 2). The specimen was sectioned to reveal a myxoid tan-yellow cut surface (figure 3). Microscopically, the specimen was uniformly hypocellular, and showed a haphazard admixture of wavy Schwann cells and collagen fibers dispersed in a mucopolysaccharide matrix (figure 4). No increased cellularity, nuclear pleomorphism, increased mitotic activity or tumor coagulative cell necrosis was identified. On immunohistochemistry the tumor was positive for S100, Neurofilament, vimentin, and negative for epithelial membrane antigen, in combination with the morphological features a diagnosis of neurofibroma was made. Pathologic examination of the skin lesion showed a malignant melanoma (6 mm in diameter) with spitzoid features: epithelioid and spindle atypical melanocytes growing in a solid, asymmetric, non-maturing pattern with deep mitotic figures (up to 3 mitotic figures/mm^2^). The individual cells displayed nuclear pleomorphism with prominent nucleoli and the epithelioid forms showed abundant cytoplasm (Figures 5, 6, 7, 8). Immunohistochemically, both the spindle and epithelioid cells showed strong and diffuse immunoreactivity for S100 and HMB-45. The proliferative index of the tumor was 30–40% as assessed with the immunohistochemical marker ki-67. This lesion was at a Clark's level IV and at a depth of 2.1 mm. Growth phase was vertical and ulceration was absent. A few tumor-infiltrating lymphocytes were present and there was no definitive evidence of regression.

## Discussion

The potential association between NF-1 and malignant melanoma has been the source of controversy in the medical literature. The common neural crest origin of these conditions has provided an attractive framework for this discussion. However, it is unclear whether patients with NF-1 have an inherently higher propensity to develop malignant melanomas than the general population. In a follow-up study of 70 NF-1 patients reported to the Swedish Cancer registry, 24% of the 70 patients developed 19 malignancies, only 1 of which was a melanoma [19]. However, the precise prevalence of melanomas in NF-1 patients is largely unknown. Most melanomas that arise in the setting of NF-1 are ocular. In a recent literature review, Honavar *et al *[20] identified only 19 reported cases overall. Cutaneous and anorectal melanomas have also been rarely reported in patients with NF-1 [21-27]. The rarity of this association given the frequency of NF-1 (1 in 3000) suggests that the probability of NF-1 patients developing malignant melanoma is no more than the general population. However, this needs to be tested in a rigorous population-based study. Ishii *et al *[21] recently reported a loss of heterozygosity (LOH) at the NF-1 gene in an anal melanoma occurring in an NF-1 patient. This suggests that the well-known Knudson's two-hit hypothesis may be operational, and that somatic loss of the second allele of the putative tumor suppressor function of the NF-1 gene causes development of this particular somatic malignancy. Again, more cases need to be studied to exclude the possibility that LOH for NF-1 occuring as a late event in the tumorigenesis of sporadic melanomas.

Our case provides another framework for the discussion of the potential association between NF-1 and malignant melanoma. Our patient has no evidence of either of the neurofibromatosis syndromes. The rarity of the clinicopathologic features of both lesions identified in this patient (the unusual presacral location of the neurofibroma and the spitzoid melanoma) suggests that their association in this patient is coincidental, even though both are neural crest derived neoplasms. This presumption contradicts the notion that in patients with NF-1, malignant melanomas that rarely develop are part of their neurocristopathy. For residents of the United States, the lifetime probability of developing cutaneous melanoma is 1 in 55–82 (approximately 1.5%) [28]. As previously noted, NF-1 is a relatively common condition with a prevalence of 1 in 3000. Since no more than 100 cases of melanoma (all sites combined) developing in NF-1 patients have been reported, the incidence is significantly lesser than the 1.5% that can be attributed to chance alone.

Although the patient described in this report did not have characteristic clinical features of NF-1, an important possibility that requires consideration is that she has segmental NF-1. Segmental NF-1 is thought to result from a post-zygotic mutation in the NF-1 gene resulting in a somatic mosaicism [29,30]. In these patients, characteristic NF-1-associated diseases are limited to a localized part of the body [29,30]. In our patient, a presacral neurofibroma was associated with an upper thigh cutaneous melanoma, putting both lesions in the general same region, albeit without true co-localization. Additionally, melanoma is not a diagnostic criteria for NF-1, as associated lesions are required to be for the definition of segmental NF-1. The location of the current patient's neurofibroma is also somewhat unusual for segmental NF-1. In two combined series that investigated 163 patients with segmental NF-1, there was not a single case of a retroperitoneal neurofibroma [29,30]. In one of these series [29], neurofibromas alone were the most common manifestation of segmental NF-1. However, in all such cases, the neurofibromas were either dermal, on major peripheral nerve trunks or both. Although these findings argue against segmental NF-1 in the current patient, the possibility certainly remains. Thus, the findings in this case should be viewed within the context of that possibility.

In conclusion, we report here the previously unreported synchronous diagnosis of a presacral neurofibroma and a spitzoid malignant melanoma in a patient without NF-1. Furthermore, the finding of a sporadic neurofibroma and malignant melanoma occurring in a patient without NF-1 lends credence to the view that when these lesions occur in patients with NF-1, the association may be coincidental.

## Competing interests

None declared.

## Authors' contributions

OF and DH made substantial contributions to the intellectual content of the paper. Both co-wrote the manuscript. Both the authors have seen the final version of the manuscript and approved it for publication.

## References

[B1] Von Deimling A, Foster R, Krone W, Kleihues P, Cavenee WK (2000). Neurofibromatosis type 1. World Health Organization Classification of Tumours: Pathology and Genetics of Tumours of the Nervous System.

[B2] Burger PC, Scheithauer BW, Vogel FS (2002). Surgical Pathology of the Nervous system and its coverings.

[B3] Feldenzer JA, McGauley JL, McGillicuddy JE (1989). Sacral and presacral tumors: problems in diagnosis and management. Neurosurgery.

[B4] Toporas M, Rusu E, Blechner M (1981). [Presacral retrorectal neurofibroma]. Rev Chir Oncol Radiol O R L Oftazmol Stomatol Chir.

[B5] Andress MR, Thomas ML (1971). Presacral neurofibroma demonstrated angiographically. Australas Radiol.

[B6] Mainetti JM, Ichcovich MN, Ayarragaray AC (1971). Presacral neurofibroma. Prensa Med Argent.

[B7] Delgaudio A (1964). Presacral retroperitoneal neurofibroma simulating an aorto-iliac aneurysm. Minerva Chir.

[B8] Nucci RC, Beyer FD (1962). Retroperitoneal and presacral neurofibroma. A case report. Obstet Gynecol.

[B9] Goldenberg IS, Alderman DB (1958). Neurofibroma following presacral neurectomy. Am J Obstet Gynecol.

[B10] Hudson OC, Ross ST (1955). Presacral neurofibroma. Am J Surg.

[B11] Bass JC, Korobkin M, Francis IR, Ellis JH, Cohan RH (1994). Retroperitoneal plexiform neurofibromas: CT findings. AJR Am J Roentgenol.

[B12] Handa VL, Jain K, McCue K, Schneider PD (2001). Posthysterectomy vault eversion with a large retroperitoneal mass. Int Urogynecol J Pelvic Floor Dysfunct.

[B13] Topsakal C, Erol FS, Ozercan I, Murat A, Gurates B (2001). Presacral solitary giant neurofibroma without neurofibromatosis type-1 presenting as a pelvic mass-a case report. Neurol Med Chir (Tokyo).

[B14] Argyrakis A, Teichmann A, Kuhn W (1985). Solitary neurofibroma of the lumbosacral plexus. J Neurol Neurosurg Psychiatry.

[B15] Chopra JS, Chander K, Kak VK (1972). Solitary plexiform neurofibroma at an unusual site. Report of a case in the lumbar region. Neurol India.

[B16] Hunter VP, Burke TW, Crooks LA (1988). Retroperitoneal nerve sheath tumors: an unusual cause of pelvic mass. Obstet Gynecol.

[B17] Spitz S (1991). Melanomas of childhood. 1948. CA Cancer J Clin.

[B18] Mones JM, Ackerman AB (2004). "Atypical" Spitz's nevus, "malignant" Spitz's nevus, and "metastasizing" Spitz's nevus: A critique in historical perspective of three concepts flawed fatally. Am J Dermatopathol.

[B19] Zöller MET, Rembeck B, Odén A, Samuelsson M, Angervall L (1997). Malignant and benign tumors in patients with neurofibromatosis type 1 in a defined Swedish population. Cancer.

[B20] Honavar SG, Singh AD, Shields CL, Shields JA, Eagle RC (2000). Iris melanoma in a patient with neurofibromatosis. Surv Opthalmol.

[B21] Ishii S, Han S, Shiiba K, Mizoi T, Okabe M, Horii A, Nagura H, Matsuno S, Sasaki I (2001). Allelic loss of the NF-1 gene in anal malignant melanoma in a patient with neurofibromatosis type 1. Int J Clin Oncol.

[B22] Silverman JF, Blahove M, Collins JL, Norris HT (1988). Cutaneous malignant melanoma in a black patient with neurofibromatosis (von Recklinghausen's disease). Am J Dermatopathol.

[B23] Mastrangelo MJ, Goepp CE, Patel YA, Clark WH (1979). Cutaneous melanoma in a patient with neurofibromatosis. Arch Dermatol.

[B24] Gallino G, Belli F, Tragni G, Ferro F, Massone PP, Ditto A, eo E, Cascinelli N (2000). Association between cutaneous melanoma and neurofibromatosis type 1: analysis of three clinical cases and review if the literature. Tumori.

[B25] Garcia-Casasola G, Casado A, Ciguenza R, Gonzalez Larriba JL, Alvarez-Sala JL (1992). Rectal melanoma and von Recklinghausen's disease. Rev Clin Esp.

[B26] Ben-Izhak O, Groisman GM (1995). Anal malignant melanoma and soft-tissue malignant fibrous histiocytoma in neurofibromatosis type 1. Arch Pathol Lab Med.

[B27] Karakayali G, Gunger E, Lenk N, Gur G, Kacar A, Alli N (1999). Neurofibromatosis and cutaneous melanoma: coincidence or association. J Eur Acad Dermatol Venerol.

[B28] Jemal A, Tiwari RC, Murray T, Ghafor A, Samuels A, Ward E, Feuer EJ, Thun MJ (2004). Cancer statistics 2004. CA Cancer J Clin.

[B29] Ruggieri M, Huson SM (2001). The clinical and diagnostic implications of mosaicism in the neurofibromatoses. Neurology.

[B30] Listernick R, Mancini AJ, Charrow J (2003). Segmental neurofibromatosis in childhood. Am J Med Genet.

